# A Non Membrane-Targeted Human Soluble CD59 Attenuates Choroidal Neovascularization in a Model of Age Related Macular Degeneration

**DOI:** 10.1371/journal.pone.0019078

**Published:** 2011-04-28

**Authors:** Siobhan M. Cashman, Kasmir Ramo, Rajendra Kumar-Singh

**Affiliations:** Department of Ophthalmology, Tufts University School of Medicine, Boston, Massachusetts, United States of America; Consiglio Nazionale delle Ricerche (CNR), Italy

## Abstract

Age related macular degeneration (AMD) is the most common cause of blindness amongst the elderly. Approximately 10% of AMD patients suffer from an advanced form of AMD characterized by choroidal neovascularization (CNV). Recent evidence implicates a significant role for complement in the pathogenesis of AMD. Activation of complement terminates in the incorporation of the membrane attack complex (MAC) in biological membranes and subsequent cell lysis. Elevated levels of MAC have been documented on choroidal blood vessels and retinal pigment epithelium (RPE) of AMD patients. CD59 is a naturally occurring membrane bound inhibitor of MAC formation. Previously we have shown that membrane bound human CD59 delivered to the RPE cells of mice via an adenovirus vector can protect those cells from human complement mediated lysis *ex vivo*. However, application of those observations to choroidal blood vessels are limited because protection from MAC- mediated lysis was restricted only to the cells originally transduced by the vector. Here we demonstrate that *subretinal* delivery of an adenovirus vector expressing a transgene for a soluble non-membrane binding form of human CD59 can attenuate the formation of laser-induced choroidal neovascularization and murine MAC formation in mice even when the region of vector delivery is distal to the site of laser induced CNV. Furthermore, this same recombinant transgene delivered to the *intravitreal* space of mice by an adeno-associated virus vector (AAV) can also attenuate laser-induced CNV. To our knowledge, this is the first demonstration of a non-membrane targeting CD59 having biological potency in any animal model of disease *in vivo*. We propose that the above approaches warrant further exploration as potential approaches for alleviating complement mediated damage to ocular tissues in AMD.

## Introduction

Age related macular degeneration (AMD) is the leading cause of central vision loss and blindness amongst the elderly in the developed world. Approximately 10% of AMD patients suffer from the exudative or wet form of AMD that is characterized by choroidal neovascularization (CNV) and macular edema. The remaining 90% of patients suffer from the nonexudative or dry form of AMD characterized by the deposition of *drusen* or lipoproteinaceous deposits between the retinal pigment epithelium (RPE) and Bruch's membrane. A subset of advanced AMD patients suffer from geographic atrophy (GA), characterized by a loss of RPE cells and subsequent retinal degeneration (reviewed in [1]). In general, wet AMD is the major contributor to vision loss and this form of AMD can be treated effectively with FDA-approved vascular endothelial growth factor antibody *ranibizumab* [2]. However, this current standard of care does not block the progression of dry AMD. Hence, there is currently no effective treatment available for 90% of AMD patients.

Recent evidence strongly implicates a role for complement in the progression of AMD. Specifically, sequence polymorphisms in complement activators and complement inhibitors have been associated with AMD (reviewed in [3]). The terminal step of complement activation involves the incorporation of the membrane attack complex (MAC) in the cell membrane that leads to their lysis [4], [5].

One hypothesis of AMD pathogenesis is that bystander complement-mediated damage to host tissues essential in maintaining healthy vision, such as the RPE or choroidal blood vessels leads to GA or CNV. Support for this hypothesis comes from the observation of elevated levels of MAC on the RPE as well as choroidal blood vessels of AMD patients [6], [7]. Sub-lytic levels of MAC are known to signal growth factor release from endothelial cells [8] and to increase mitogenesis [9], suggestive of a possible role for MAC in the formation of CNV or the transition of dry to wet AMD. MAC has been documented to be elevated in the laser photocoagulation model of wet AMD and attenuation of MAC inhibits the formation of CNV [10]. Collectively, these data are in support of the hypothesis that MAC plays a role in AMD.

CD59, also known as *protectin* or *membrane inhibitor of reactive lysis* is a glycosylphosphatidyl inositol (GPI)-anchored membrane inhibitor of MAC formation on the plasma membrane [11], [12]. CD59 functions by binding the C5b678 terminal complement protein complex and preventing the incorporation of the multiple C9 molecules required to complete the formation of a pore in the cell membrane [13]. Recombinant soluble forms of CD59 (sCD59) from which the GPI membrane anchor has been deleted have been previously described [14], [15]. However, relative to membrane-bound CD59, sCD59 has proven to be a poor inhibitor of MAC both *in vitro* and *in vivo*. Studies designed to measure the MAC-inhibitory capacity of sCD59 have found that in a ‘reactive lysis’ system where purified components are used to assemble the MAC on targets, sCD59 can inhibit MAC formation. However, in the presence of serum, this capacity is significantly diminished [15], [16]. Moreover, *in vivo*, the small size of sCD59 results in rapid clearance in the kidney. To our knowledge, there is thus far no clear reported evidence of a non-membrane binding CD59 being able to inhibit MAC formation *in vivo*.

Those early studies on the lack of efficacy of sCD59 *in vivo* prompted the development of soluble recombinant membrane-targeted forms of CD59 that were targeted to membranes via a membrane address tag [17]. Such novel molecules have substantially higher levels of activity relative to membrane-free sCD59 and have been found to be efficacious in a number of animal models of disease [17], [18], [19]. Previously, we have shown that expression of a GPI-anchored human CD59 expressed on murine RPE *in vivo* protects those cells from human complement mediated attack *ex vivo* [20]. However, only the cells that synthesized and expressed the CD59 molecule on their surface were protected from complement-mediated damage. While an important first step, this approach was limiting and did not lend itself to application towards inhibition of choroidal neovascularization or targeting of blood vessels- a tissue intimately implicated in wet AMD. Ideally, a subset of cells ought to be utilized as ‘factories’ for local production and secretion of a soluble CD59 in order to confer protection to a large number of ocular cells including the RPE and choroidal blood vessels.

In this study we revisit the use of a non-membrane binding sCD59 as an inhibitor of MAC formation *in vivo* using a gene therapy approach. We believe that the ocular environment in combination with a gene therapy approach provides unique opportunities for the use of sCD59 despite previous discouraging data on the use of this molecule *in vivo*. We elected to test our approach of inhibiting MAC in a previously well-described model of wet AMD i.e, choroidal neovascularization (CNV) induced by photocoagulation of the Bruch's membrane. Our results indicate that sCD59 without the need for any membrane targeting can significantly attenuate MAC formation and reduce CNV *in vivo*. We propose that use of sCD59 delivered by a gene therapy approach deserves further study as a potential therapy for both the wet as well as dry forms of AMD.

## Results

### Soluble human CD59 is correctly processed and secreted *in vitro*

**Figure 1:**
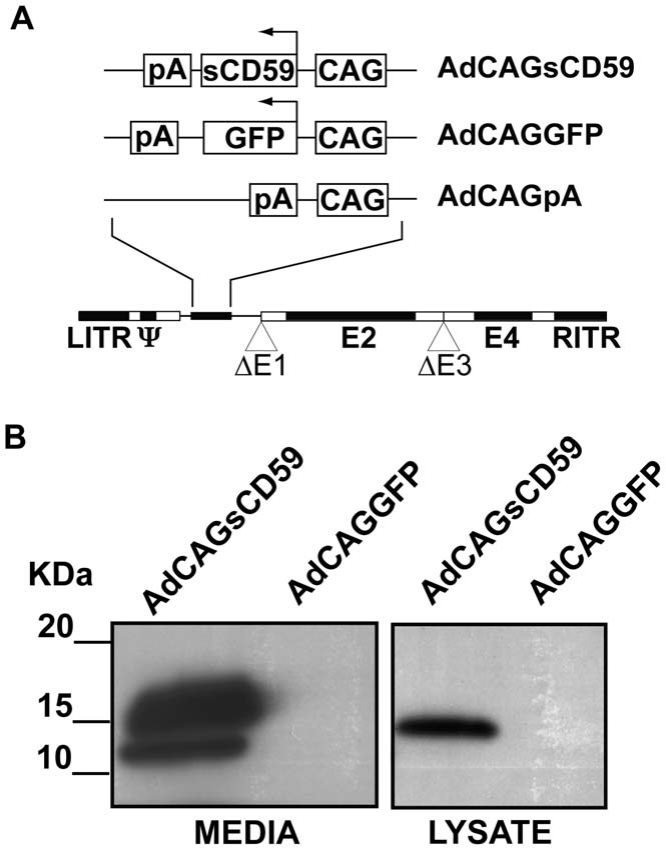
sCD59 is expressed and efficiently secreted from adenovirus *in vitro.* (A) Expression cassettes for both sCD59 and GFP or without a transgene were cloned into the deleted E1 region of an E1/E3-deleted adenovirus. (B) ARPE-19 cells infected with AdCAGsCD59 express sCD59 and exhibit robust secretion into the media. CAG, chicken b-actin promoter; pA, polyA; LITR/RITR, left/right inverted terminal repeat; ¥, packaging signal; ΔE1/E3, deleted early regions 1/3; E2/E4, early regions 2/4.

A recombinant human adenovirus serotype 5 containing an expression cassette for human soluble CD59 (sCD59), which lacks the C-terminal 26 amino acids encoding the signal sequence for attachment of the GPI anchor, was generated (AdCAGsCD59, Figure 1A) essentially as described by us previously [20]. An adenovirus expressing a GFP (AdCAGGFP) and an adenovirus without a transgene (AdCAGpA) were also used in this study and have been described previously [20]. AdCAGsCD59 was examined for efficient expression and secretion of sCD59 by infection of ARPE-19 cells with either AdCAGsCD59 or a control virus, AdCAGGFP. After infection, cell lysate and media were harvested and analyzed by western blot. Media from cells infected with AdCAGsCD59 confirmed the expression and secretion of sCD59 (Figure 1B), a protein consisting of a number of N-glycosylated forms of approximately 14–18 kDa. The lysate from these infected cells exhibited a discrete band at approximately 15 kDa. As expected, no sCD59 was detected in the lysate or media from AdCAGGFP-infected ARPE-19 cells.

### sCD59 confers protection from human serum-mediated cell lysis

**Figure 2:**
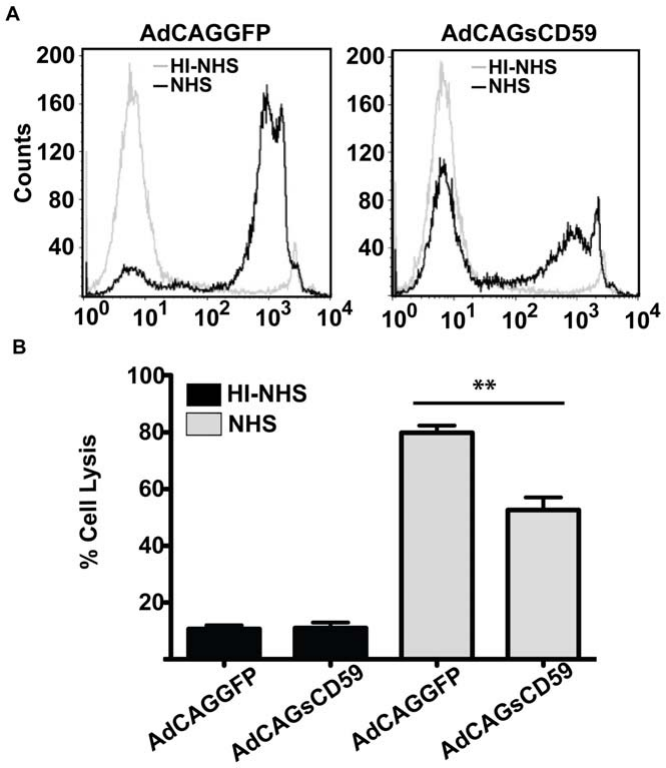
AdCAGsCD59-conditioned media confers significant protection from human serum-mediated cell lysis. (A) A representative FACS histogram for hepa-1c1c7 cells treated with either NHS or HI-NHS in media conditioned with either AdCAGGFP or AdCAGsCD59. (B) While no significant difference in cell lysis was observed in hepa-1c1c7 cells treated with HI-NHS in media conditioned with either AdCAGGFP or AdCAGsCD59, a significant (34.08±6.40%, **p<0.01) reduction was observed in cell lysis in hepa-1c1c7 cells treated with NHS in media conditioned with AdCAGsCD59 relative to those cells treated with NHS in media conditioned with the control virus, AdCAGGFP. HI-NHS, heat-inactivated normal human serum; NHS, normal human serum. n = 4.

Murine Hepa-1c1c7 cells were incubated at a final concentration of 1% normal human serum (NHS) in media pre-conditioned by media from either AdCAGsCD59 or AdCAGGFP- infected ARPE-19 cells. As a control, a similar set of studies were performed using heat inactivated NHS (HI-NHS). One hour post-incubation, Hepa-1c1c7 cells were analysed for lysis by flow cytometry for uptake of propidium iodide. A representative histogram of cell lysis is presented in Figure 2. Whereas lysis of Hepa-1c1c7 cells treated with NHS and AdCAGGFP-conditioned media was 79.87±2.54% (Figure 2B), lysis of Hepa-1c1c7 cells treated with NHS and AdCAGsCD59-conditioned media was 52.66±4.43%. A small amount of cell lysis was observed in Hepa-1c1c7 cells treated with HI-NHS and either the AdCAGGFP-conditioned media (10.68±1.27%) or the AdCAGsCD59-conditioned media (11.03±1.92%), which could be the result of cell manipulation during the experiment (Figure 2B). In conclusion, AdCAGsCD59-conditioned media conferred a significant (34.08±6.40%, p<0.01) protection against cell lysis mediated by complement in NHS.

### Adenovirus does not influence the size of laser-induced CNV

**Figure 3:**
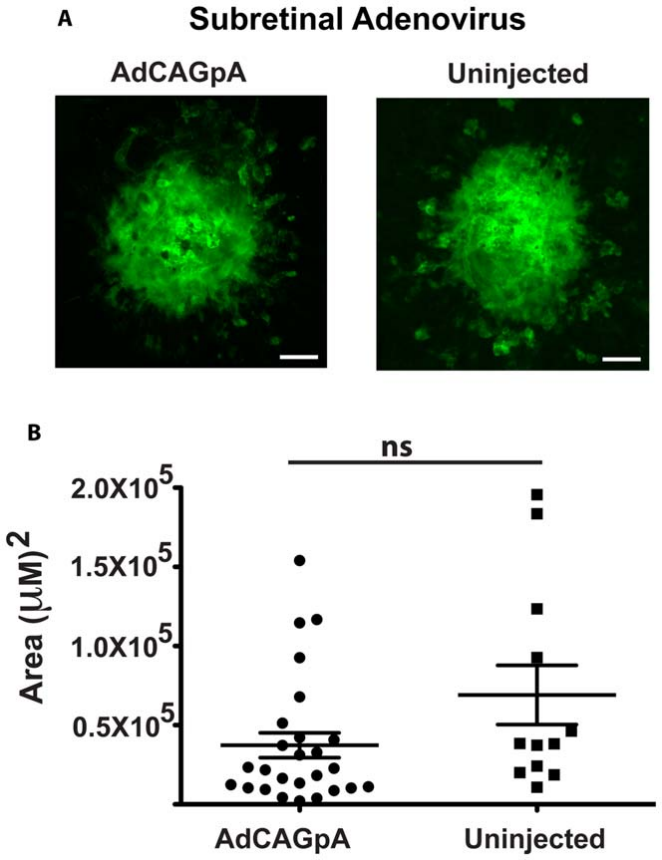
Delivery of adenovirus to mouse subretinal space does not affect size of laser-induced CNV. Representative micrographs of FITC-GSL I stained laser-induced CNV spots from each of AdCAGpA-injected and uninjected eyecups. No significant difference was observed in the size of spots induced by laser in AdCAGpA-injected relative to uninjected eyes (p>0.5). n = 12 spots/5 eyes (uninjected), n = 26 spots/9 eyes (AdCAGpA); ns, not significant. Scale bar = 50 µm.

Adenovirus has been previously shown to induce complement activation *in vivo* [21], [22]. To examine whether ocular delivery of an adenovirus into a mouse would affect the amount of choroidal neovascularization (CNV) induced by laser burn, we injected a control virus, AdCAGpA (spiked in a 1∶10 ratio with AdCAGGFP to localize the injection site) into the subretinal space of 6 week-old C57Bl6/J mice. 72 hours post-injection, both AdCAGpA-injected and uninjected mice were subjected to three laser burns per eye and CNV allowed to develop for 7 days. Eyes were harvested, cornea, lens, and retina removed, and RPE/choroid flatmounts stained with a FITC-conjugated lectin specific for endothelial cells, GSL I, isolectin B4. A representative micrograph of a CNV spot is presented for both AdCAGpA-injected and uninjected eyes (Figure 3A). The average size of a laser CNV spot in AdCAGpA-injected eyecups was 3.73±0.78×10^4^ µm^2^, while the average spot size in the uninjected eyecups was 6.91±1.88×10^4^ µm^2^. We therefore conclude that there was no significant difference in CNV spot size between AdCAGpA-injected and uninjected eyecups (p>0.5, Figure 3B), and that adenovirus delivery into the murine subretinal space does not significantly affect the size of the CNV induced by a laser burn.

### *Subretinal* delivery of an adenovirus expressing sCD59 leads to a reduction in laser-induced CNV

**Figure 4:**
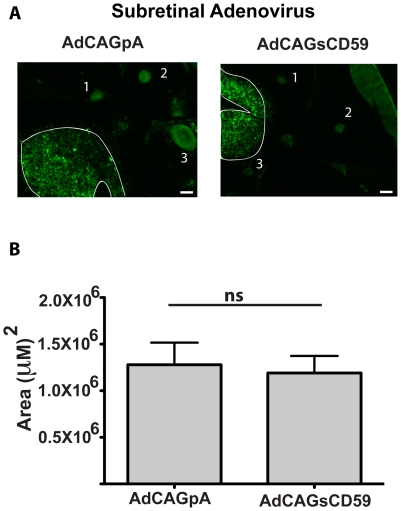
AdCAGpA and AdCAGsCD59 coinjected with AdCAGGFP result in equivalent levels of transduction and at sites distal to the sites of laser burn. Representative micrographs showing RPE/choroid flatmounts transduced with AdCAGGFP:AdCAGpA (1∶10) and AdCAGGFP:AdCAGsCD59 (1∶10). The region of transduction is demarcated and each of the three laser spots labeled 1–3. No significant difference in area of transduction was observed between AdCAGpA-injected and AdCAGsCD59-injected eyecups. n = 10 eyes (AdCAGsCD59), n = 8 eyes (AdCAGpA). ns, not significant. Scale bar = 200 µm.

**Figure 5:**
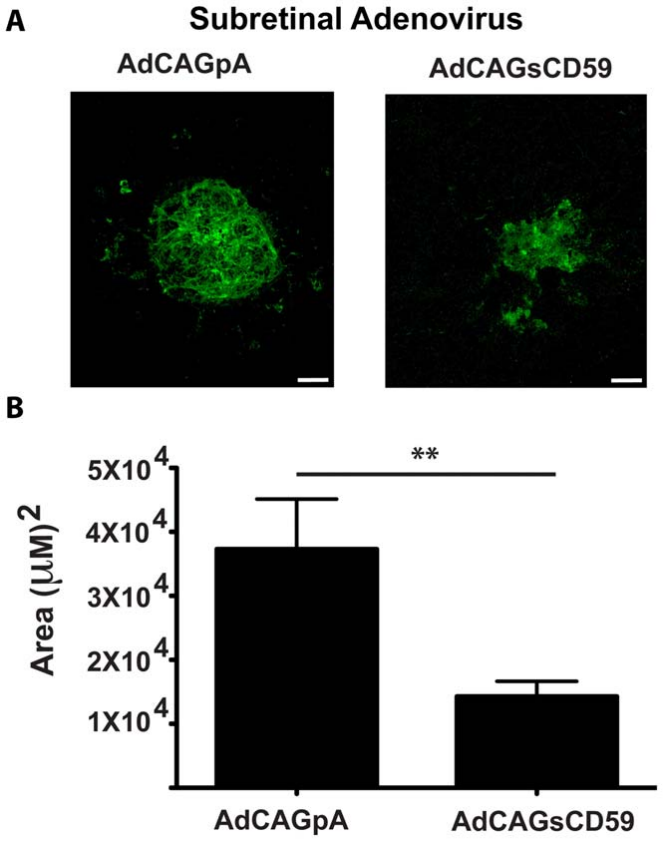
AdCAGsCD59 delivered to murine RPE results in a significant reduction in laser-induced CNV. Representative micrographs showing FITC-GSL I stained laser-induced CNV spots from eyes injected with either AdCAGpA or AdCAGsCD59. AdCAGsCD59 transduction of murine RPE results in a 61.7±19.9% reduction in size of CNV spot relative to eyes injected with AdCAGpA (**p<0.01). n = 33 spots/12 eyes (AdCAGsCD59), n = 26 spots/9 eyes (AdCAGpA). Scale bar = 50 µm.

Each of AdCAGpA and AdCAGsCD59 (spiked in a 1∶10 ratio with AdCAGGFP) were injected into the subretinal space of mice and eyes treated with laser as described above. 7 days post-laser, RPE/choroid flatmounts were stained with GSL I. A micrograph showing the region of adenovirus transduction relative to the sites of laser burn for each of AdCAGpA and AdCAGsCD59 is shown (Figure 4A). No significant difference in the area of transduction (p>0.05) was observed between AdCAGpA-injected eyecups (1.28±0.24×10^6^ µm^2^) relative to AdCAGsCD59-injected eyecups (1.19±0.18×10^6^ µm^2^, Figure 4B). CNV spots from these eyes were imaged by confocal fluorescent microscopy and the size of CNV spots measured. A representative micrograph of a CNV spot from an AdCAGpA-injected and AdCAGsCD59-injected eyecup is shown (Figure 5A). While the average size of CNV spots from AdCAGpA-injected eyecups was 3.73±0.78×10^4^ µm^2^, the average spot size from AdCAGsCD59-injected eyecups was 1.43±0.24×10^4^ µm^2^ (Figure 5B). This resulted in a significant (61.7±19.9%, p<0.01) reduction in size of CNV spot by AdCAGsCD59, indicating that delivery of AdCAGsCD59 to murine RPE by subretinal injection confers a significant protection against laser-induced CNV.

### *Subretinal* delivery of an adenovirus expressing sCD59 leads to a reduction in MAC

**Figure 6:**
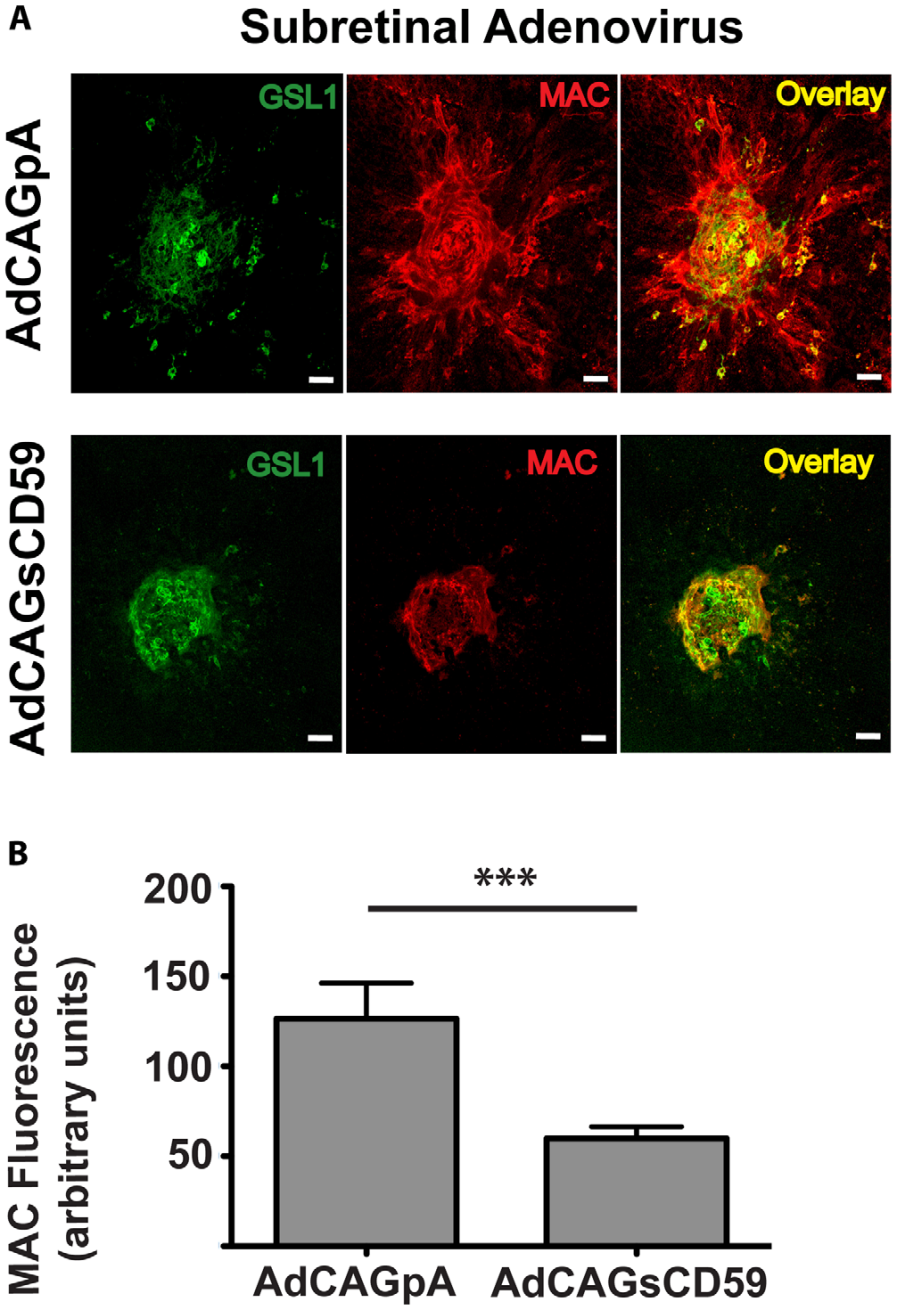
AdCAGsCD59 results in a significant reduction in MAC deposition at the site of laser-induced CNV. Representative micrographs showing laser-induced CNV spots from eyes injected with AdCAGpA and AdCAGsCD59 and co-stained with GSL I and anti-C9 antibody (MAC). For more accurate representation of MAC staining, CNV spots of equivalent size are shown for each of AdCAGpA and AdCAGsCD59. The overlay illustrates deposition of MAC extending beyond the region staining positive for GSL I, particularly in the AdCAGpA-injected eyecup. A significant (52.5±13.4%, ***p<0.001) reduction in MAC deposition was observed in regions of laser burn in AdCAGsCD59-injected eyecups relative to those injected with AdCAGpA. n = 11 spots/4eyes (AdCAGpA), n = 20 spots/7 eyes (AdCAGsCD59). Scale bar = 50 µm.

RPE/choroidal flatmounts injected with either AdCAGpA or AdCAGsCD59 and subjected to laser burn as described above were stained with an antibody against mouse C9. MAC staining in the AdCAGpA-injected eyecups could be observed extending beyond the region of GSL I staining (5 of 11 spots, Figure 6A), while in the AdCAGsCD59-injected eyecups MAC deposition was confined to the region of GSL I staining (20 of 20 spots). This suggests that cells adjacent to the CNV spot are being protected from MAC deposition in the AdCAGsCD59-injected eyecups. Quantification of MAC staining showed a significant 52.5±13.4% (p<0.001) reduction in MAC deposition in AdCAGsCD59-injected eyecups (Figure 6B) relative to those injected with AdCAGpA.

### *Intravitreal* delivery of an adeno-associated virus expressing sCD59 leads to a reduction in laser-induced CNV

**Figure 7:**
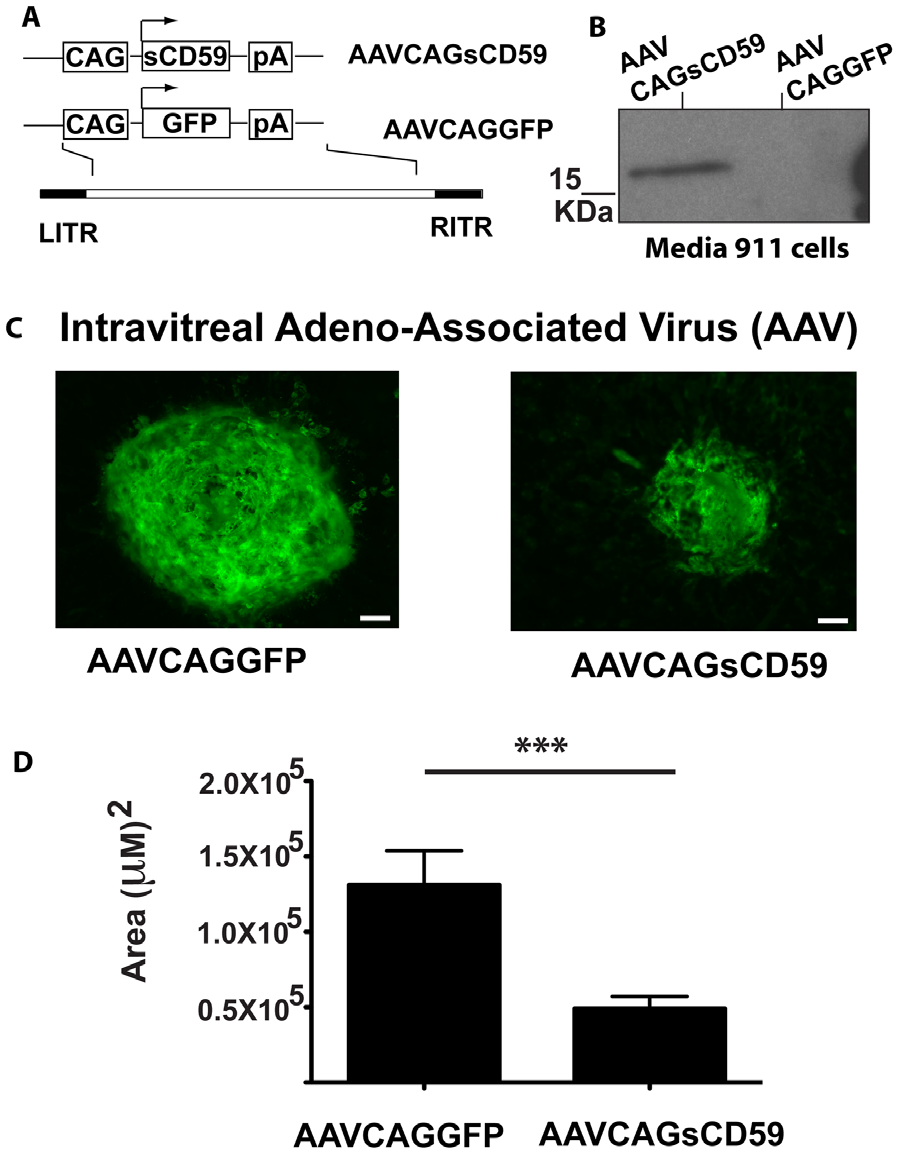
Intravitreal delivery of an AAV expressing sCD59 results in a significant protection against laser-induced CNV. (A) The expression cassette containing sCD59 under the control of the chicken β-actin promoter was cloned into an AAV serotype 2 vector. A control virus expressing GFP was also generated. (B) Western blot analysis confirms secretion of sCD59 by human embryonic retinoblasts infected with AAVCAGsCD59. (C) Representative micrographs of laser-induced CNV spots observed in AAVCAGGFP-injected and AAVCAGsCD59-injected eyecups. (D) A significant 62.6±16.9% (***p<0.001) reduction in size of CNV was observed in AAVCAGsCD59-injected eyecups relative to those injected with AAVCAGGFP. n = 42 spots/11 eyes (AAVCAGGFP), n = 53 spots/14 eyes (AAVCAGsCD59). Scale bar = 50 µm.

In contrast to subretinal injection, intravitreal injection is a much more widely used approach for ocular drug delivery and is used routinely for administration of anti-VEGF treatments for wet AMD. Adeno-associated virus (AAV) serotype 2 has been shown to be safe when administered to the vitreous of non-human primates [23]. The sCD59 or GFP expression cassettes were cloned into an AAV-2 vector and are referred to as AAVCAGsCD59 and AAVCAGGFP respectively (Figure 7A). Expression of sCD59 was confirmed by western blot analysis of infected human embryonic retinoblasts (Figure 7B). A unique band at approximately 15 kDa was observed in the media of cells infected with AAVCAGsCD59 and as expected, absent from the media of cells infected with the control virus AAVCAGGFP. AAVCAGsCD59 (spiked 1∶10 with AAVCAGGFP) was injected into the vitreous of the eyes of 6–8 week old mice. Control eyes were similarly injected with AAVCAGGFP alone but both eyes received the same total number of AAV particles. Between 12 and 19 days post-injection, the eyes of injected mice were subjected to laser burn and eyes harvested 7 days post-laser. A representative image of a laser-induced CNV from each of AAVCAGGFP-injected and AAVCAGsCD59-injected eyecups is shown (Figure 7C), in which a significant 62.6±16.9% (p<0.001) reduction was observed in AAVCAGsCD59-injected eyecups relative to those injected with the control virus, AAVCAGGFP (figure 7D). The average size of CNV from AAVCAGGFP-injected eyes was 1.31±0.23×10^5^ µm^2^, while that of the AAVCAGsCD59-injected eyecups was 0.49±0.08×10^5^ µm^2^. It is noteworthy that CNV lesions in AAV-injected eyes were larger than in those injected with adenovirus. We consider that this is possibly due to the increased number of spots (4) generated in AAV-injected eyes versus adenovirus-injected eyes (3) as it is likely that the release of growth factors from each lesion may influence the growth of adjacent lesions.

## Discussion

Substantial genetic, biochemical as well as histological evidence now exists that links complement activation with the pathogenesis of AMD. Based on this cumulative evidence, a number of clinical trials examining the safety and efficacy of various inhibitors of complement are now under way. These trials are targeting either complement component C3 via a small peptide (compstatin/POT-4), complement component C5 via an antibody *(eculizumab)* or aptamer (ARC1905), or Factor D via an antibody (TNX-234) (reviewed in [24]). The complement system plays an essential role in innate immunity against microbes and orchestrates immunological and inflammatory processes. Naturally occurring deficiencies of specific complement components highlight the need for a fully functional complement system. For example, C3 or C5 deficiency has previously been associated specifically with Neisseria and meningococcal meningitis infections respectively [25], [26], [27]. Hence, blocking the activity of C3 or C5 may potentially expose individuals to bacterial infections. Furthermore, a block in C3 or C5 activation would also result in a block in production of anaphylatoxins C3a and C5a that act directly on neutrophils and monocytes to speed up the phagocytosis of pathogens or function in the activation of mast cells and antibody recruitment. Complement is also involved in diverse processes such as synapse formation, angiogenesis, mobilization of hematopoietic stem-progenitor cells, tissue regeneration and lipid metabolism (reviewed in [28]). Accordingly, it has recently been suggested that loss of synapses via complement may play a role in glaucoma [29]. These diverse functions of complement further highlight the need for an intact complement system. In view of these considerations, it may be advantageous to leave as much of the complement system intact as possible and attenuate only the terminal step in complement mediated damage, i.e. the formation of MAC. Blocking the formation of MAC via CD59 satisfies these criteria, notwithstanding the possibility that pathogens may be capable of recruiting sCD59 to their surfaces and evading complement mediated lysis.

Peptides and antibodies have limited half-lives *in vivo* and need to be readministered on a regular basis. For example, wet AMD patients receive intraocular *ranibizumab* antibody injections every 4 to 6 weeks. This exposes patients to complications associated with endophthalmitis. Whereas the incidence of endophthalmitis is relatively low (0.16% per dose) in the presence of a robust immune system [30], this rate is likely to substantially increase due to the cumulative effect of an attenuated complement system and serial injections over many years for chronic diseases such as AMD. Hence, frequent injection of complement inhibitors into AMD eyes may not be desirable. Limiting the frequency of administration may be satisfied via a gene therapy approach as we have done in this study.

Viral vectors such as adenovirus have previously enabled transgene expression *in vivo* in mice for their lifetime [31]. AAV vectors have enabled transgene expression in dogs for more than 7 years (reviewed in [32]). In humans, AAV has been found to have therapeutic transgene expression for over 3.7 years [33]- the longest time periods studied. We selected adenovirus as a gene delivery vector because we have previously found it to be an efficient vector for delivery of transgenes to ocular tissues [20], [34], [35] as well as the observation that adenovirus has been found to be safe in several ocular gene therapy trials [36], [37], [38]. Adenovirus vectors can be designed to permit long-term transgene expression and the technology for scaled production of such vectors is well described [31]. Nonetheless, we demonstrated that our observations may be extended to AAV vectors as well. Such vectors have previously been shown to be safe for use in humans and are generally considered less immunogenic than adenovirus vectors. While not directly examined in this study, it is likely that sCD59 delivered to AMD eyes via an AAV vector would permit long-term transgene expression. Moreover, given that sCD59 is already found naturally [39] in ocular tissues (albeit not likely at the levels following recombinant transgene expression) the prospect of an immune response against sCD59 is further diminished.

Previously, we have shown that GPI-anchored human CD59 delivered via an adenovirus vector *in vivo* can protect murine RPE from human serum-mediated lysis *ex vivo* [20]. However, as anticipated, protection was conferred only upon the cells that were transduced by the vector. In human AMD eyes, MAC is found not only on the RPE but also on the choroidal blood vessels and Bruch's membrane. Since delivery of the vector to all of these cells and membranes via an intraocular approach is not technically feasible, we embarked upon testing the efficacy of a soluble non-membrane associated version of CD59 (sCD59). Prior studies have found that sCD59 is not efficacious *in vivo* and hence a variety of approaches employing recombinant sCD59 with membrane targeting addresses or sCD59 fusion proteins have been described [17]. The structure of the ocular compartments provides unique barriers when targeting cells in the outer ocular tissues (RPE, choroid) via subretinal or intravitreal delivery. Any membrane-targeting molecule injected into the subretinal space is not likely to reach the choroidal blood vessels due to sequestration by the RPE membrane. Similarly, any membrane-targeting molecule injected into the vitreous is likely to be sequestered by the cells lining the vitreous cavity. Hence, a diffusible soluble CD59 might be necessary to overcome the above barriers.

One complication and apparent limitation of our studies is due to the small size of the mouse eye that sometimes leads to traction and partial detachment of the retina during intravitreal injection. Such detachments are self-resolving within 24 hours. Delivery of AAV expressing GFP into the vitreous of mice sometimes led to transduction of the RPE, presumably through retinal detachment during the injection procedure. These observations highlight the limitations of the small mouse eye and studies in larger eyes, for example dogs, pigs or non-human primates are warranted to extend the observations associated with intravitreal delivery of AAV for delivery of transgene products to the outer retina. Recent studies performed in non-human primates receiving an intravitreal injection of an AAV vector expressing modified soluble VEGF receptor (sFLT01) have observed a reduction in laser induced CNV [40]. Given that sCD59 has a significantly lower molecular weight than sFLT01, it is likely that it too can permeate the non-human primate retina, but this remains to be demonstrated. Previous studies in mice in which membrane-targeting murine or rat CD59 have been injected into the vitreous did not include an approach to detect retinal detachment occurring during the intravitreal injection and hence unintended access to the subretinal space may have occurred [10], [19].

In summary, we have shown for the first time that membrane targeting of sCD59 is not required to inhibit MAC formation *in vivo*. We have demonstrated that human sCD59 delivered via a gene therapy approach can attenuate CNV in mice using either an adenovirus vector injected subretinally or an AAV vector injected intravitreally. We have also demonstrated that sCD59 can reduce the extent of MAC deposition on laser-induced CNV spots. Our results suggest that further studies are warranted towards the development of sCD59 as a therapy for the wet as well as dry forms of AMD and possibly other ocular diseases of complement.

## Methods

### Generation of adenovirus and AAV constructs

Human CD59 lacking the sequence encoding the C-terminal 26 amino acids was amplified by PCR using the primers CD59XH2F (5′-CCCCCTCGAGTGGACAATCAC AATGGG-3′) and CD59StopEV-R (5′-TAAGGAGATATCTTAATTTTCAAGCTGTT CGTTA-3′) from the cDNA obtained from American Type Culture Collection. The reverse primer introduces a stop codon after asparagine 77. The PCR product was digested with XhoI (New England Biolabs) and EcoRV (New England Biolabs) and ligated with a similarly-digested pShCAG [20] pShCAG was recombined with pAdEasy-1 and used to generate the adenovirus, AdCAGsCD59, as previously described [20]. The control viruses, AdCAGGFP and AdCAGpA have been described previously [20]. AAVCAGsCD59 was generated by insertion of the CAGsCD59pA expression cassette (as employed in the adenovirus construct) into pAAV-MCS (Stratagene) to generate pAAVCAGsCD59 (further cloning details available upon request). The AAVCAGsCD59 virus was produced and purified by the UMass Vector Core Facility, using AAV serotype 2 capsid proteins. An AAV-2 vector expressing GFP from a chicken b-actin promoter (AAVCAGGFP) was purchased from the vector core facility.

### Western analysis

Adenovirus: ARPE-19 cells (ATCC) were infected with either AdCAGsCD59 or AdCAGGFP at an MOI 1000 in DMEM (Invitrogen). Cell lysate and media were loaded on a 10% Tris-HCl gel (Bio-Rad Criterion). The membrane was probed with an anti-CD59 antibody ((N-20) sc-7077, Santa Cruz), followed by a HRP-bovine anti-goat antibody (Jackson ImmunoResearch). AAV: Human embryonic retinoblasts [41] were infected at an MOI 7000 in DMEM. Four days post-infection, cell lysate and media was analyzed as for adenovirus.

### NHS-mediated cell lysis

ARPE-19 cells were infected with either AdCAGsCD59 or AdCAGGFP at an MOI 1000 in serum-free DMEM without phenol red and maintained at 37°C/5%CO_2_. Three days post-infection, media was collected and centrifuged at 2500RPM to remove any cells. Hepa-1c1c7 cells were resuspended in media conditioned by infected ARPE-19 cells and either normal human serum (NHS) or heat-inactivated NHS (final concentration of 1%) and incubated at 37°C with constant gentle rotatory motion. One hour post-incubation, propidium iodide was added to cells (2 mg/ml) and uptake analyzed by flow cytometry. 2.5×10^4^ cells were counted per sample (FACSCalibur, Becton Dickinson) and analyzed (CellQuest Pro software, Becton Dickinson).

### Intraocular injections

The use of animals in this work was in accordance with the Statement for the Use of Animals in Ophthalmic and Vision Research, set out by the Association for Research in Vision and Ophthalmology. This study was approved by Tufts University Institutional Animal Care and Use Committee (IACUC) protocol B2009-03 and Tufts University Institutional Biosafety Committee registration 2008-BRIA27. C57BL/6J mice were purchased from Jackson Laboratories (Bar Harbor, ME), bred and maintained in a 12-hour light–dark cycle and cared for in accordance with federal, state and local regulations. Mice were anesthetized by intraperitoneal injection of 0.1 ml/10 g of xylazine (10 mg/ml)/ketamine (1 mg/ml). Subretinal or intravitreal injections were performed with a 32 G needle attached to a 5 µl glass syringe (Hamilton, Reno, NV) by a trans-scleral trans-choroidal approach to deliver 2.4×10^7^ total particles of adenovirus or 8×10^9^ genome copies of AAV. Both eyes of each mouse were injected with the same virus.

### Laser Induced Choroidal Neovascularization

Mice were anesthetized as described above, and pupils dilated with a drop each of 2.5% phenylephrine HCl/1% Tropicamide (Bausch and Lomb). A drop of Hypromellose/Gonak (2.5%, Akorn, Inc) was applied to the cornea and the back of the eye viewed with the aid of a microscope and cover slip held on the cornea. An argon laser (532 nm) set at 100 milliseconds and 150 mW was used to generate three laser spots (75 µm) in each eye (adenovirus) or four laser spots per eye (AAV).

### Staining and imaging of RPE/choroid flatmounts

Mice were sacrificed by CO_2_ inhalation and eyes harvested 7 days post-laser treatment. Optic nerve, cornea, lens, iris and retina were removed and the RPE/choroid/sclera (referred to as RPE/choroid) fixed overnight in 4% paraformaldehyde. RPE/choroid tissue was incubated in 2.5 mg/ml BSA in PBS for 30 mins at 37°C, followed by 100 mg/ml FITC-conjugated Griffonia Simplicifolia Lectin I (GSL I, isolectin B4, Vector Labs) in PBS for 1 hour at 37°C. For MAC staining, RPE/choroid was incubated with 6% normal goat serum in 0.3% Triton-PBS (PBST) for 30 mins at RT, followed by a 1∶200 dilution of rabbit anti-mouse C9 antibody (kind gift of B. Paul Morgan) in PBST for 2.5 hours at RT and a 1∶400 dilution of CY3-conjugated goat anti-rabbit (Jackson ImmunoResearch) at RT for 1 hour. Flatmounts were imaged using both conventional microscopy using an Olympus IX51 with appropriate filters, a Retiga 2000R FAST camera and QCapture Pro 5.0 (QImaging, British Columbia, Canada) software and by confocal microscopy using a Leica TPS SP2.

### Measurement of laser spots, adenoviral transduction, and intensity of MAC staining

Area of both GSL I-stained spots and adenoviral transduction was measured using ImageJ software (developed by Wayne Rasband, National Institutes of Health, Bethesda, MD; available at http://rsb.info.nih.gov/ij/index.html). Intensity of MAC staining was determined by measurement of pixel intensity within the area of the laser spot using ImageJ. Background pixel intensity of each image was subtracted from pixel intensity of the spot. Area and intensity measurements were analyzed with Prism 5 (GraphPad Software, Inc) using an unpaired student's *t*-test. All data is presented as Mean±SEM.
